# Contribution of families using the GenIDA database to the description of MED13L syndrome and literature review

**DOI:** 10.1186/s11689-025-09618-4

**Published:** 2025-05-19

**Authors:** Roseline Caumes, Pauline Burger, Jean-Louis Mandel, Hélène Béhal, Jamal Ghoumid, Thomas Smol

**Affiliations:** 1 Univ. Lille, ULR7364 – RADEME – Maladies RAres du DEveloppement embryonnaire et du Métabolisme, Lille, France; 2 CHU Lille Clinique de Génétique, Lille, France; 3 Department of Neurogenetics and Translational Medicine, Institute of Genetics and Molecular and Cellular Biology (IGBMC), Université de Strasbourg, INSERM U1258, CNRS UMR7104, Illkirch, France; 4 University of Strasbourg, Institute for Advanced Studies (USIAS), Strasbourg, France; 5 CHU Lille, Unité Statistique, Evaluation Economique et Data Management (SEED), Lille, France; 6 CHU Lille, Institut de Génétique Médicale, avenue Oscar Lambret, Lille, F-59000 France

**Keywords:** MED13L, Intellectual disability, Family, Patient registry

## Abstract

The GenIDA project aims to improve the understanding and management of rare genetic forms of intellectual disability by fostering collaboration among patients, caregivers, healthcare professionals, and research professionals. Clinical data is provided by patients’ families *via* a structured questionnaire to identify medically relevant insights and better understand the natural history of rare diseases. This study focused on MED13L syndrome, analyzing data from 41 patients in the GenIDA database and comparing it with 102 cases from the scientific literature and 6 new descriptions of patients from our medical center.

The GenIDA series confirmed the key features of MED13L syndrome, including global developmental delay, poor speech, intellectual disability, and cardiac defects (OMIM #616789), at frequencies similar to those reported in the literature. The GenIDA series identified a higher prevalence of visual impairment (76%) and highlighted under-recognized musculoskeletal issues, such as foot deformities, which had previously received little attention. This study highlights the value of family-reported data in describing the full phenotype of rare syndromes. A comprehensive review of published cases showed that patients with missense variants have more severe impairments, including increased cardiac defects, global developmental delay, and a higher incidence of epilepsy, than patients with premature truncated variants.

These findings highlight the importance of family involvement in rare disease research and the need for further studies to explore genotype-phenotype correlations to improve patient care and outcomes.

**Supplementary Information:**

The online version contains supplementary material available at 10.1186/s11689-025-09618-4.

## Introduction

Created in 2014 and accessible online since 2016 (https://genida.unistra.fr/), the international project GenIDA is promoted by INSERM (French National Institute of Health and Research) and aims to improve the knowledge and management of rare genetic forms of intellectual disabilities (ID). Families are asked to fill in a 46-item online form with mainly closed multiple choice questions, which, after synthesis and statistical analysis, can be accessed by a medical expert in the patient’s rare disease [1]. More than 2100 patients from 60 different countries are currently enrolled. Among the different series, the MED13L syndrome series included 44 participants as of July 2022, with 41 completed questionnaires [1]. Indeed, with the development of sequencing (exome or genome) approaches for patients with ID or global developmental delay (GDD), *MED13L* appears to be one of the most frequently affected genes in series of patients with ID/GDD [2, 3].

Pathogenic variants within the *MED13L* gene cause MED13L syndrome (MIM #616789), described as a global developmental delay in motor and language acquisition, with ID severity ranging from mild to severe. Some malformations may be associated, particularly cardiac and skeletal malformations. There are currently around 100 patients described in the scientific literature, either in case reports or in the form of series of patients who have undergone gene panel, exome or genome analysis, with brief clinical descriptions [4–6]. Several publications have noted varying degrees of severity in the phenotype of patients, with some pathogenic missense variants being more severe [4, 5, 7]. However, there is no precise information on the evolution and frequency of other symptoms, and therefore on the follow-up that should be proposed to patients.

Here, we aimed to consider the experience of parents in describing the MED13L syndrome, compare it with information from the medical literature, and then to compare phenotypes previously reported in the literature and assocaited with predicted truncating variants (PTV) vs. missense variants.

## Methods

### Ethical approval

The parents or legal guardians of the patients registered in the GenIDA database gave informed consent for the data collected in the questionnaire to be used in scientific studies and agreed to be contacted with additional questions. The study has been declared to the “Commission Nationale Informatique et Libertés” (CNIL) on 27/11/2015, number 1907912v0. It has been reviewed and approved by the Ethics Evaluation Committee of INSERM (IORG0003254, FWA00005831). This study, including all procedures, complied with the Declaration of Helsinki and French laws and regulations. Six novel descriptions were included. Their information was aggregated within the overall statistical analysis. No identifiable clinical histories or specific pathogenic variant coordinates are described, ensuring patient confidentiality. The patients were informed during consultations at the University Hospital about the potential reuse of their data, and no objections were raised. Our study adheres to the recommendations of our Data Protection Officer (DPO) and the Health Data Warehouse (EDS INCLUDE).

### Data collection procedures

Information from 41 families enrolled in the MED13L syndrome series in the GenIDA database were analyzed. Of the 46 items, only those completed by more than two-thirds of the families were retained in the study to ensure a minimum of 30 patients per item, consistent with more robust statistical approaches. The questionnaire included closed questions in a multiple-choice format and open comments to specify answers. Due to European regulations, patients’ molecular data are not included in the questionnaire to preserve their anonymity [1].

A comprehensive PubMed search was conducted using the free text term ‘MED13L’ over the period of 2004-March 2022. Additional records were identified using PubMed Medical Subject Headings (MeSH). For large series of patients with ID/GDD, we selected publications from the Human Gene Mutation Database (HGMD) [8]. We grouped patients described more than once and excluded those without a clinical description other than ID/GDD, often from large series of patients who benefited from genome-wide genetic analysis. Thus, the published clinical features of 102 patients with either pathogenic or likely pathogenic *MED13L* variants according to the ACMG criteria were analyzed [9]. We had descriptions of 6 new patients for a total of 108 patients. The clinical data of the six new patients were collected at our center. Written informed consent was obtained from the parents. Of these patients, 24 had missense and non-frameshift variants, whereas 84 exhibited loss-of-function variants: 20 with deletions (whole gene or intragenic), intragenic duplications [3], or single variants (non-sense (19 patients) or frameshift variants (33 patients)), and 9 with splicing variants.

To enable a standardized comparison of previously published data, we chose to use the Human Phenotype Ontology (HPO) database to describe each feature of the phenotype. We coded only the items that were explicitly studied or considered in each publication, regardless of whether they were present or absent in patients [10].

### Statistical analyses

Categorical variables are described by frequencies and percentages. Quantitative variables are expressed as mean and standard deviation. Patients’ characteristics were compared between two groups (first between scientific literature and GenIDA database; second between missense and loss-of-function -LOF- variants) using the Chi-square or Fisher exact test in case of categorical variables; by using Student t-test for quantitative variables. Statistical analyses are performed using SAS software (SAS Institute version 9.4). Statistical testing was performed at a two-tailed α level of 0.05.

## Results

### General overview

We analyzed data from 41 patients in the GenIDA series, with an average age of 14 years [range: 4 years-51 years]. Predominantly, patients were from France (48.5%), followed by the United States (20%) and other European countries. These data were compared with information on 108 patients (sex ratio = 1) with MED13L syndrome extracted from either exome series [11–26] or case reports [4–6, 27–38]. The mean age of patients in the literature was 9.7 years [range: 7 months-39 years]. Common challenges reported by families included visual impairment (76%, 29/38), intellectual disability (68%, 28/41), musculoskeletal issues (66%, 27/41), and walking difficulties (61%, 25/41). Literature frequently highlighted delayed speech and language development (91%, 91/106), delayed gross motor skills (81%, 86/106), and global developmental delay (73%, 77/106). While facial features were not assessed in GenIDA, morphological traits extracted from literature indicated recurrent elements, such as a bulbous nasal tip (80%, 47/59), open mouth (85%, 47/55), and up-slanted palpebral fissure (51% 31/63). Most patients had head circumferences within the normal range (88%) (Table 1).

**Table 1:** Characteristics of patients according to literature and GenIDA series

Features	GenIDA patients (%)	Literature patients (%)
Mean age (years) [range]	14 [4–51]	9.7 [0.5–39]
Visual impairment	29/38 (76.0)	42/46 (91.3)
Intellectual disability	28/41 (68.0)	86/86 (100)
Absent speech (if patient > 2years old)	12/37 (32.4)	30/72 (41.7)
Isolated words	7/41 (17.1)	Not evaluated
Elocution abnormality	17/41 (41.4)	Not evaluated
Nonverbal communication	14/41 (34.1)	Not evaluated
Cardiac defect	5/37 (13.5)	22/78 (28.2)
Seizures	4/37 (10.8)	16/67 (23.9)
Autism	9/28 (32.1)	25/65 (38.5)
Hearing defect	13/41 (32.0)	42/46 (91.0)
Vision impairment	32/41 (78.0)	42/45 (93.3)
Hypermetropia	21/41 (51.2)	12/45 (26.6)
Strabismus	16/41 (39.0)	16/45 (35.5)
Dental abnormality	16/41 (39.0)	5/5 (100)
Musculoskeletal issues	27/41 (66.0)	5/5 (100)
Walking difficulties	25/41 (61.0)	Not available
Feeding disorders	20/42 (47.0)	23/30 (79.0)

### Neurodevelopmental evaluation

Intellectual disability and/or developmental delay are key features of MED13L syndrome in the literature. However, the severity of ID was only specified in 54 out of 86 patients with detailed information. Among these, ID was classified as mild in 4% of patients (2/54), moderate in 70% (38/54), and severe in 26% (14/54). Additionally, all reported patients exhibited speech delay, and among those older than 2 years, 30 patients/72 (41%) were reported to have no speech at the time of description. Lack of speech was significantly more frequent in patients with missense pathogenic variants than in those with loss-of-function variants (*p* = 0.0127). Parental ID evaluations in the GenIDA series were consistent with those reported in the literature. ID severity was classified in 30 of 41 patients with 23% classified as mild, 47% as moderate, and 30% as severe to profound (Table 1). It is important to note that these classifications were based on subjective parent reports rather than formal IQ testing and specific score cutoffs. Speech evaluations were detailed in the GenIDA database, thereby providing more accurate developmental information. The first words were acquired at a median of 24 months. Among patients over 2 years of age, 12 out of 37 (32%) were reported to have no language, and 7 out of 41 (17%) communicated using isolated words. Non-verbal communication was noted for only 14 patients and used by 11 of them.

### Motor evaluation

There was no significant difference in the mean age of walking between patients in the GenIDA series (*n* = 41) and those in the literature series (*n* = 65) with ages of 28 and 27 (SD = 6.59) months, respectively. The mean age at onset of sitting was 12 months in the GenIDA series, and this milestone is not often reported in publications. Parents frequently noted walking difficulties in patients from the GenIDA series after 2 years of age, with 58% (24/41) exhibiting issues such as an ‘unstable walk’, a sense of ‘fatigability’, and ‘hypotonia’. These items were consistent with published data: hypotonia was present in 87% of patients (65/74), abnormal gait was described in 39 patients. Of these patients, 20 out of 39 have an ataxic gait. Severe motor delay was reported in 15.5% of patients in the literature who did not achieve walking independently (*n* = 12), although 2 of them were under 2 years old (Table 1). Severe motor delay was significantly more frequent in patients with missense pathogenic variants than in those with PTV variants (Table 2; *p* = 0.0141).

**Table 2:** Characteristics of patients according to missense and loss-of-function

Features	Missense patients *N* = 24 (%)	PTV patients *N* = 84 (%)	*p*-value	Significance
Mild intellectual disability	0/11 (0.0)	3/45 (6.7)	0.390	No
Moderate intellectual disability	5/11 (45.5)	34/45 (75.6)	0.055	No
Severe intellectual disability	6/11 (54.6)	8/45 (17.8)	0.012	Yes
Unacquired walking after 2 years of age	6/16 (37.5)	6/61 (9.8)	0.014	Yes
Age of walking achievement (months)	28.4 ± 4.1	27.2 ± 7.0	0.210	No
Absent speech	11/16 (68.8)	19/56 (33.9)	0.012	Yes
Autism	9/16 (56.3)	16/49 (32.7)	0.092	No
Seizure	8/15 (53.3)	8/52 (15.4)	0.005	Yes
Gait ataxia	4/8 (50.0)	16/31 (51.6)	1.000	No
Abnormal brain imaging	12/17 (70.6)	27/46 (58.7)	0.388	No
Hypotonia	14/17 (82.4)	51/57 (89.5)	0.420	No
Visual abnormality	12/13 (92.3)	30/33 (90.9)	0.880	No
Abnormality of cardiovascular system morphology	9/18 (50.0)	13/60 (21.7)	0.019	Yes
Bulbous nose	8/11 (72.7)	39/48 (81.3)	0.680	No
Up-slanted palpebral fissure	8/13 (61.5)	24/50 (48.0)	0.384	No
Open mouth	10/13 (76.9)	37/42 (88.1)	0.38	No

*PTV* Premature Truncating Variants

### Musculoskeletal abnormalities

Musculoskeletal abnormalities were observed in 65% of patients in the GenIDA series (27/41). These included ‘scoliosis’ in 15% (6/41), ‘anomalies of the lower limbs’ in 25% (10/41), and ‘upper limb particularities’ in 7% (3/41). Regarding lower limb anomalies, parents frequently mentioned ‘club feet (5)’ or ‘flat feet (6)’ in their comments. In the literature, abnormalities of the extremitiesare rarely reported in detail. Among the 53 patients with such abnormalities, 22 exhibited foot malformations, including 11 cases of *metatarsus adductus*, 11 cases of *talipes equinovarus*, and 10 cases of toe abnormalities without further specification. Upper limb malformations were noted in only 25 patients, and detailed descriptions were lacking. The most commonly described hand anomalies were clinodactyly (5 cases) and camptodactyly (3 cases).

### Neurologic and psychiatric comorbidities

In the GenIDA series, ‘epilepsy’ was reported in 6 out of 41 patients (14%), whereas epilepsy was absent in 30 patients. Information regarding epilepsy status was not provided for 5 patients as the item was left unfilled. Unfortunately, the questionnaire and accompanying comments did not include details on therapeutics or the natural history of the disease. Epilepsy status was documented in 67 patients in the literature, with seizures observed in 24% of the patients (16/67). Epilepsy was significant more frequent in patients with missense pathogenic variants than in those with loss-of-function variants (53.3% vs. 15.4%, *p* = 0.005). Furthermore, only 4 EEGs were mentioned, and the types of seizures were defined in 6 patients, including absence seizures in 2 cases, febrile seizure for 2 and focal epilepsy in 2 cases (Table 2).

The frequency of reported autism was not significantly different between the two series, with 32% (9/28) in GenIDA and 38% (25/65) in the literature. Behavioral difficulties were comprehensively detailed in the GenIDA database, providing more accurate information from the parents. They described behavioral troubles in 60% of the patients (25/41), including mild to severe ‘attentional disorder’ in 56% (23/41), ‘intolerance to frustration’ in 51% (21/41), ‘restricted interests’ in 46% (19/41), and ‘anxiety’ in 41% (17/25). Additionally, ‘aggressiveness’ was reported in 41% (17/25) of the cases. Sleeping disorders were observed in 22% of patients (9/41), although specific details were not provided.

In the literature, brain imaging was detailed for 63 patients and was found to be normal for 37% of them. Most of abnormalities were nonspecific, including white matter abnormalities (17%, 11/63), ventriculomegaly (17%, 11/63), abnormal corpus callosum morphology (14%, 9/63), or Dandy-Walker malformation (3%, 2/63). In GenIDA, 93% of patients (38/41) underwent brain imaging, without further detailed findings noted.

### Cardiac defects

Initially, congenital heart defects were considered a significant aspect of MED13L syndrome and were therefore frequently investigated. Cardiac findings were documented for 77 patients in the literature, and 28% of them (22/78) specified the cardiac problem. These included patent foramen ovale (12%, *n* = 9), tetralogy of Fallot (4%, *n* = 3), patent ductus arteriosus (3%, *n* = 2), and ventricular septal defects (3%, *n* = 2). A lower percentage was observed in the GenIDA series, with cardiac involvement reported for 14% of the patients (5/37), although no detailed description was provided.

### Sensorineural evaluation

In the GenIDA series, visual disorders were described in 78% of patients (32/41), with ‘hypermetropia’ in 51% (21/41) and ‘strabismus’ in 39% (16/41). Observations were consistent with those reported in the literature, with strabismus in 36% (16/45), and hypermetropia in 27% (12/45). There was no statistical difference between the number of patients with missense pathogenic variants and those with loss-of-function variants and visual impairment.

‘Hearing problems’ were reported by parents in 32% of patients (13/41). Recurrent Ear-Nose-Throat infections were reported in 16 patients of whom, 6 required tympanic aerators. Hearing impairment status was assessed in only 15 patients in the literature, with 9 of them presenting with impairment (i.e. 14% of the patients described). Recurrent respiratory infections and otitis media were rarely reported (3 and 4 patients, respectively).

### Other comorbidities

Dental anomalies were described in only 5 patients from the literature, without recurrent features (carious teeth, hypoplasia of the dental arch, supernumerary maxillary incisor, incisor macrodontia). Parental assessment in GenIDA reported a higher number of ‘dental anomalies’, observed in 39% of patients (16/41) with only a few further descriptions. In the literature, cleft palate was described in 7 patients, including 4 patients who had Pierre-Robin Sequence.

Other features that were not documented in the literature but reported by parents included ‘eczema’ in 19% (8/41). Feeding difficulties were reported in 48% of patients (20/41), including ‘chewing problems’, ‘impulsivity and/or food selectivity’. Digestive problems were reported in 39% of patients in the GenIDA series (16/41), including ‘constipation’ (12/15) or ‘repeated reflux/vomiting’ (3/13).

## Discussion

GenIDA is an international online research project initiated to better characterize the clinical manifestations and natural history of genetic forms of intellectual disability with or without autism or epilepsy by encouraging collaboration between patients, caregivers and healthcare professionals [1]. The patient’s family provides and updates clinical information through a structured questionnaire. These data are analyzed to uncover new medically relevant insights that may benefit both families and healthcare professionals managing the condition. The current questionnaire includes 46 multiple-choice questions covering various aspects, such as physical characteristics, cognitive and behavioral traits, and the presence of neurological disorders or issues affecting major physiological functions (e.g., cardiac, renal) (Supplemental Data 1) [1]. Although feedback on the ease of use of the surveys was not collected in this study, future evaluations could incorporate items to assess parental experiences and challenges in completing the surveys. GenIDA data were previously used for Koolen-De Vries syndrome and confirmed that the day-to-day experiences of families dealing with this type of condition lead parents to be strong partners in delineation of rare syndromes [39].

Although the difference in average ages between the GenIDA series and the publication may introduce potential bias, with the publication having a higher average age, this did not affect the strength of the parents’ descriptions of MED13L syndrome. Thus, the involvement of families in the description of MED13L syndrome through the GenIDA initiative has enabled us to confirm most previously observed comorbidities [4–6]. Consistent with the findings of the analysis of Ruault et al. for *DDX3X*-related intellectual disability, this finding highlights the value of GenIDA in improving screening for associated features and comorbidities [39]. The GenIDA study on MED13L syndrome confirmed a high prevalence of visual impairment, observed in 76% of patients, which is in contrast with the existing literature reporting this condition in only about half of patients [4–6]. Hearing and visual screening are recommended to improve the management of these patients. Taken together, parental evaluation of patients, combined with a review of the literature, could contribute to a comprehensive clinical evaluation of MED13L syndrome and support the growth of larger, global research initiatives [40].

The cardinal signs of MED13L syndrome, i.e., global developmental delay, poor speech, intellectual disability, and cardiac defects were described with similar frequencies, with no significant difference between the literature and the GenIDA cohort. This concordance has already been reported in the Koolen-de Vries syndrome cohort [1, 41]. Developmental delays, including an average walking age and lack of speech, were found to be equally common [4–6]. Regarding intellectual disability, few details on psychometric tests were available for patients, both in scientific publications and from families. Analysis of the two cohorts confirms that, unlike the first reported patients, cardiac defects are not predominant, either in frequency or impact, as they usually require only simple monitoring [4, 28, 29, 35, 42].

Interestingly, the family perspective can highlight some under-described aspects of the phenotype. Musculoskeletal issues have been reported in 66% of patients in the GenIDA series. Re-analysis of the published patients allowed us to identify the descriptions of metatarsus adductus in 11 patients [6, 30, 31, 35], and talipes equinovarus in 10 patients [4, 7, 29, 33, 37]. Thus, foot malposition is a common sign of MED13L syndrome. Although it has been described previously, it has received little attention until now. These features may be related to the expression of *MED13L* mRNA, which is ubiquitously expressed in human tissues, and has the highest levels in the cerebellum, fetal brain, and skeletal muscle [30].

As genetic data collection was not included in the GenIDA project due to European legislation, genotype/phenotype correlations were not possible to establish. However, a review of all patients described in published articles enabled us to assess the impact of different pathogenic variants, categorized as missense versus premature truncating variants [41]. We observed a statistically significant difference for some aspects of MED13L syndrome. Patients with a missense variant were more likely to have a cardiac defect, as well as more significant developmental delay, including failure to walk independently, and more frequent epilepsy. This greater impairment in patients carrying a missense variant had already been reported in two previously published MED13L-series [4, 5].

The GenIDA initiative has effectively increased the understanding of MED13L syndrome through comprehensive data collection and strong collaboration between patients, caregivers, and healthcare professionals. This study confirmed a higher prevalence of visual impairment, highlighted under-recognized musculoskeletal problems, and emphasized the importance of family involvement in identifying the full phenotype. Although genetic data collection was limited, a review of published cases showed that patients with missense variants have more severe impairments, highlighting the need for further research into genotype-phenotype correlations.

## Supplementary Information

Supplementary Material 1: Supplemental Data 1. English version of the GenIDA questionnaire, detailing all 46 items.

## Data Availability

No datasets were generated or analysed during the current study.
