# Supplementary material for: Contribution of families using the GenIDA database to the description of MED13L syndrome and literature review
Source: J Neurodev Disord. 2025 May 19;17:28. doi: 10.1186/s11689-025-09618-4 (PMC12087057; doi:10.1186/s11689-025-09618-4)
Supplement: Supplementary file 1 — Supplementary Material 1: Supplemental Data 1. English version of the GenIDA questionnaire, detailing all 46 items. [file 11689_2025_9618_MOESM1_ESM.pdf]

GENIDA – Questionnaire GenIDA (20 Oct. 2016 release).

OPEN QUESTIONS

1/46. What is the major problem that affects your relative's everyday life (quality of life)?
(Even if it seems unrelated to the genetic condition.) *(Free answer)*

2/46. What is the major behavioral / cognitive problem that affects your relative's and the family life? *(Free answer)*

3/46. Did your relative suffer a major medical problem or discomfort following specific medication?
(Specify drug and length of treatment) *(Free answer)*

4/46. What are the major medical problems that occurred until now? (Indicate at what age for each) *(Free answer)*

5/46. Please state your relative's strengths *(Free answer)*

WEIGHT / HEIGHT

6/46. Your relative's weight:
Unit *(select the right unit)*
- Kilograms
- Pounds

At birth:
6m:
1y:
2y:
4y:
6y:
10y:
14y:
Current value:

7/46. Your relative's height:
Unit *(select the right unit)*
- Centimeters
- Inches

At birth:
6m:
1y:
2y:
4y:
6y:
10y:
14y:
Current value:

8/46. Head circumference: (only if it has been measured by a clinician)
Unit *(select the right unit)*
- Centimeters
- Inches

At birth:
6m:
1y:
2y:
4y:
6y:
10y:
14y:
Current value:

PREGNANCY / EARLY LIFE / PERSONAL CARE

9/46. Was there any problem during pregnancy, labour and/or delivery?
Yes / No / I don't know *(Single Choice)*

What words best describe them? *(Select one or more answers)*
Reduced fetal movement

Lack of fetal movement
Low amniotic fluid
Excessive amniotic fluid
Increased nuchal fold thickness
Abnormal ultrasound results
Caesarean section
Vacuum and/or forceps extraction
Other: *(if 'Other' is selected, please specify in free text)*

10/46 Apgar scores: (only if measured by a professional)
1 min:
5 min:
10 min:

11/46. Has your relative experienced any health problems in his/her newborn period (first four weeks)?
Yes / No / I don't know *(Single Choice)*

What words best describe them? *(Select one or more answers)*
Signs of anoxia (lack of oxygen)
Jaundice
Hypotonia at birth (muscle weakness)
Hypertonia at birth
Feeding difficulties
Other: *(if 'Other' is selected, please specify in free text)*

What treatment was performed?
Assisted ventilation
Tube feeding
Gastrostomy (tube connecting the stomach)
Jejunostomy (tube connecting the small intestine)
Other: *(if 'Other' is selected, please specify in free text)*

How long was the newborn hospitalized? *(in days)*

12/46. Developmental milestones: please mark any milestone that your relative has achieved stating his/her age (if achieved)
Sitting without support: *(In months)*
Standing independently: *(In months)*
Walking independently: *(In months)*
First words: *(In months)*

13/46. Personal care:
Age (by default current age) *(In years)*

Your relative can: *(Choose one answer for each proposition: Yes / Yes with help / No)*
Actively assist with personal care?
Wash, brush her/his teeth?
Dress her/himself?
Manage shoelaces?
Use potty / toilet for urine (daytime)
Use potty / toilet for urine (night-time)
Use potty / toilet for faeces (daytime)
Use potty / toilet for faeces (night-time)

How much help does she/he need during the day? *(Free answer)*

How much help does she/he need during the night? *(Free answer)*

INTELLECTUAL DISABILITY AND AUTISM ASPECTS
14/46. Communication ability:
Age (by default current age) *(In years)*

Does your relative communicate in any of the following ways?
With speech
Vocal sounds / noises
Gestures
With written words
With symbols / pictures
Signing
None of these choices
Other: *(if 'Other' is selected, please specify in free text)*

At what age did your relative achieve the present level of communication? *(In years)*

Please describe any difference between what your relative understands and what she/he can express:

15/46. Speech ability:
Age (by default current age) *(In years)*

Single Choice
Full correct sentences
Full sentences but often incorrect
No sentence, but single words
Difficult to understand (elocution problems)
Not able to speak

Understand what is being said?
Yes / No / I don't know *(Single Choice)*

if you choose "Not able to speak":
Non-verbal communication:
Good expressive non-verbal communication
Poor non-verbal communication
No communication at all

Is your relative's speech likely to be understood by: *(Select one or more answers)*
The family
Others who meet your child for the first time
Neither the family, nor others

16/46. Reading ability:
Age (by default current age) *(In years)*

Single Choice
Good
Poor
Non reading

Has been diagnosed with dyslexia or dyscalculia? Yes / No / I don't know *(Single Choice)*

17/46. Writing ability:
Age (by default current age) *(In years)*

Single Choice
Good
Poor
Not able to write

18/46. Education:
Does your relative has a statement of special educational need (or equivalent)? Yes / No / I don't know *(Single Choice)*

What in summary does it say? *(Free answer)*

What learning support does your relative receive? *(Free answer – e.g., one to one assistance in school, etc.)*

19/46. Diagnosis of intellectual disability?
Yes / No / I don't know *(Single Choice)*

What is the degree of this intellectual disability?
Mild
Moderate
Severe
Profound

20/46. Has an IQ test been performed?
Yes / No / I don't know *(Single Choice)*

Date (By default: today)

Which test? *(Free answer)*

Test result: *(Free answer)*

21/46. Diagnosis of autism? (autism spectrum disorder)
Yes / No / I don't know *(Single Choice)*

22/46. Has an Autism test been performed?
Yes / No / I don't know *(Single Choice)*

Date (By default: today)

Which test? *(Free answer)*

Test result: *(Free answer)*

23/46. Behavior problems:
Age (by default current age) *(In years)*

Yes / No / I don't know *(Single Choice)*

What words best describe them? *(Select one answer for each proposition: Nonexistent / Mild / Moderate / Major)*
Aggressive
Self aggressiveness (self mutilation)
Impulsivity
Hyperactive
Attention deficit
Shy
Anxious
Depressive tendencies
Restricted interests
Repetitive behavior / stereotypes
Obsessions
Phobias
Diagnosed as schizophrenia
Other: *(if 'Other' is selected, please specify in free text)*

24/46. Feeding problems:
Age (by default current age) *(In years)*

Yes / No / I don't know *(Single Choice)*

What words best describe them? *(Select one or more answers)*
Eats too much
Craves for food
Eats only very restricted food
Does not want to eat (anorexia)
Other: *(if 'Other' is selected, please specify in free text)*

How do you evaluate its (or their) seriousness?
Major
Moderate
Mild

25/46 How would you describe your relative's sociability?
Age (mandatory) *(In years)*

In each of these four cases, select : Very sociable / Average sociability / Little sociability / No interaction
With familiar adults
With unfamiliar adults
With familiar children
With unfamiliar children

PHYSICAL AND NEUROLOGICAL PROBLEMS
26/46. Walking problems:
Age (by default current age) *(In years)*

Yes / No / I don't know *(Single Choice)*

What words best describe them? *(Select one or more answers)*
Never learned to walk
Learned to walk but stopped walking
Walk for a limited period, on a limited distance
Walk with cane or other aid
Unstable walk
Other: *(if 'Other' is selected, please specify in free text)*

How do you evaluate its (or their) seriousness?
Major
Moderate
Mild

27/46. Tremor:
Age (by default current age) *(In years)*

Yes / No / I don't know *(Single Choice)*

(if 'yes', specify in free answer)

How do you evaluate its (or their) seriousness?
Major
Moderate
Mild

28/46. Other mouvement disorders:
Age (by default current age) *(In years)*

Yes / No / I don't know *(Single Choice)*

What words best describe them? *(Select one answer for each proposition: No / Mild / Moderate / Major / I don't know)*
Abnormal movements or tics
Fine motor skills problems
Spastic paraplegia
Ataxia
Other: *(if 'Other' is selected, please specify in free text)*

29/46. Epilepsy: has your relative had any seizures (fits)?
Yes / No / I don't know *(Single Choice)*

Date (By default: today)

End of period (optional: fill only to define a period)

Does this epilepsy require constant medication? Yes / No

If you have select "yes" at the previous question: What is the name of the drug / molecule ? (If other drugs has been previously tested, please answer this question again and adjust the period of traitment)

What is (or was) the effectiveness of the drug ?
No effect
Very good
Good
Little effect
Not well tolerated
Major or significant adverse effect
Other: *(if 'Other' is selected, please specify in free text)*

Is this epilepsy pharmaco-resistant? Yes / No

Please describe the type(s) of epilepsy and its seriousness: *(Select one answer for each proposition: No / Mild / Moderate / Major / I don't know)*
Infantile spasms
Febrile convulsions (fever fit)
Absence seizures
"Grand mal" seizures (Tonic-clonic seizures)
Atonic (drop) seizures
Simple partial seizures (focal seizures)
Complex partial seizures (focal seizures)
Nocturnal seizures
Lennox-Gastaut syndrome
Unknown type of seizures
Other: *(if 'Other' is selected, please specify in free text)*

30/46. Has your child had any imaging or other investigations of the head / brain? (brain scanner / MRI / etc.)
Age (by default current age) *(In years)*

Yes / No / I don't know *(Single Choice)*

Type of scan / analysis and results *(Free answer)*

SENSORY PROBLEMS
31/46. Vision problems:
Age (by default current age) *(In years)*

Yes / No / I don't know *(Single Choice)*

Please describe the type(s) of problem: *(Select one answer for each proposition: Yes / No / I don't know)*
Far-sighted (hypermetropia, problems with near vision)
Short-sighted (myopia, problems with far vision)
Squint / cross eyed (strabismus)
Cataract
Nystagmus (involuntary eye movement)
Other: *(if 'Other' is selected, please specify in free text)*

Does your relative need glasses? Yes / No / I don't know *(Single Choice)*

Far-sighted lens diopter (+):

Short-sighted lens diopter (-):

32/46. Hearing problems:
Age (by default current age) *(In years)*

Yes / No / I don't know *(Single Choice)*

What words best describe them? *(Select one or more answers)*
Deafness (no devises)
Recurrent ears infections
Ear malformation
Other: *(if 'Other' is selected, please specify in free text)*

What is the seriousness of the hearing impairment?
Mild (1)
Moderate (2)
Severe (3)
Profound (4)

1. Mild: Troubles in noisy situations.
2. Moderate: Can hear and understand speech only in quiet situations.
3. Severe: Cannot hear a speech without the use of amplification.
4. Profound: Cannot hear most environmental sounds without the use of amplification.

How are the hearing problems managed? *(Select one answer for each proposition: Yes / No / I don't know)*
Hearing aids
Hearing tubes (ear or tympanostomy tubes)
Other: *(if 'Other' is selected, please specify in free text)*

33/46. Sense of smell problems:
Age (by default current age) *(In years)*

Yes / No / I don't know *(Single Choice)*

(Please specify in free text)

How do you evaluate its (or their) seriousness?
Major
Moderate
Mild

OTHER PROBLEMS
34/46. Sleeping disorders:
Age (by default current age) *(In years)*

Yes / No / I don't know *(Single Choice)*

What words best describe them?
Sleep apnea
Other: (if 'Other' is selected, please specify in free text)

How do you evaluate its (or their) seriousness?
Major
Moderate
Mild

35/46. Dental anomalies:
Age (by default current age) *(In years)*

Yes / No / I don't know *(Single Choice)*

What words best describe them? *(Select one or more answers)*
Too many teeth
Too few teeth
Frequent dental caries
Complex orthodontics
Other: *(if 'Other' is selected, please specify in free text)*

How do you evaluate its (or their) seriousness?
Major
Moderate
Mild

36/46. Oral / buccal problems:
Age (by default current age) *(In years)*

Yes / No / I don't know *(Single Choice)*

What words best describe them? *(Select one or more answers)*
Swallowing difficulties
Hypersalivation
Cleft lip
Cleft palate
Other: *(if 'Other' is selected, please specify in free text)*

How do you evaluate its (or their) seriousness?
Major
Moderate
Mild

37/46. Cardiac problems:
Age (by default current age) *(In years)*

Yes / No / I don't know *(Single Choice)*

What words best describe them? *(Select one answer for each proposition: Yes / No / I don't know)*
Cardiomyopathy
Cardiac rythms problems (abnormal EKG)
Cardiac or cardiovascular malformation
Atrial septal defect (ASD)
Ventricular septal defect (VSD)
Patent ductus arteriosus (PDA)
Pulmonary stenosis
Tetralogy of Fallot (TOF)
Bicuspid aortic valves (BAV)
Aortic aneurysm (dilatation / broadening)
Other: *(if 'Other' is selected, please specify in free text)*

What treatment / surgery (if any) has been needed or is anticipated?

38/46. Vascular problems:
Age (by default current age) *(In years)*

Yes / No / I don't know *(Single Choice)*

What words best describe them? *(Select one or more answers)*
Hypertension
Hypotension
Coagulation problems
Other: *(if 'Other' is selected, please specify in free text)*

How do you evaluate its (or their) seriousness?
Major
Moderate
Mild

39/46. Respiratory and pulmonary problems:
Age (by default current age) *(In years)*

Yes / No / I don't know *(Single Choice)*

What words best describe them? *(Select one answer for each proposition: Yes / No / I don't know)*
Asthma
Hay fever (Allergic rhinitis)
Respiratory rate problem
Pulmonary function problem
Laryngomalacia
Tracheomalacia
Other: *(if 'Other' is selected, please specify in free text)*

How do you evaluate its (or their) seriousness?
Major
Moderate
Mild

40/46. Digestive problems:
Age (by default current age) *(In years)*

Yes / No / I don't know *(Single Choice)*

What words best describe them? *(Select one answer for each proposition: No / Mild / Moderate / Major / I don't know)*
Regurgitation
Constipation
Diarrhea
Repeated vomiting episodes
Intestinal obstruction
Other: *(if 'Other' is selected, please specify in free text)*

41/46. Renal / Kidney, bladder and urogenital system problems:
Age (by default current age) *(In years)*

Yes / No / I don't know *(Single Choice)*

What words best describe them? *(Select one answer for each proposition: Yes / No / I don't know)*
Renal malformation (e.g. horseshoe kidney)
Insufficient renal function
Urinary system malformation
Frequent urinary tract infection
Vesico-ureteral reflux (VUR)
Undescended testicles (cryptorchidism)
Other: *(if 'Other' is selected, please specify in free text)*

Regularly followed by a specialist: Yes / No

Underwent surgery: Yes / No

Takes medication: Yes / No

What is the name of the drug / molecule ? (If other drugs has been previously tested, please answer this question again and adjust the period of traitment)

42/46. Musculo-skeletal problems:
Age (by default current age) *(In years)*

Yes / No / I don't know *(Single Choice)*

What words best describe them? *(Select one answer for each proposition: Yes / No / I don't know)*
Malformation of the vertebral column
Scoliosis
Kyphosis (Hunchback / hump back)
Fragility (ex: broken bones)
Malformation of the upper limb
Malformation of the lower limb
Skull anomaly or malformation
Pectus excavatum (hollowed chest)
Pectus carinatum (pigeon chest)
Hip dysplasia (deformation or misalignment of the hip joint)
Pes planus (flat feet)
Pes cavum (a high arch of the feet)
Club foot (congenital talipes equinovarus CTEV)
Contractures (permanent shortening of a muscle or joint)
Joint laxity (ligamentous laxity, looseness)
Other: *(if 'Other' is selected, please specify in free text)*

Did your relative went through any surgery? *(if 'yes', specify)*

43/46. Skin, nails and hair problems:
Age (by default current age) *(In years)*

What words best describe them? *(Select one answer for each proposition: Yes / No / I don't know)*
High number of moles
Skin pigmentation disorder (e.g. hypopigmentation)
Eczema, psoriasis or other skin inflammatory problems
Non-inflammatory anomalies of the skin (scar, photosensibility, etc.)
Abnormal nails (soft, brittle, unusual shape, etc.)
Hair color different from parents and siblings
Fine wispy hair (unlike parents or siblings)
Thick hair (unlike parents or siblings)
Other: *(if 'Other' is selected, please specify in free text)*

How do you evaluate its (or their) seriousness?
Major
Moderate

Mild

44/46. Endrocine and metabolic systems problems:
Age (by default current age) *(In years)*

Yes / No / I don't know *(Single Choice)*

What words best describe them? *(Select one or more answers)*
Hyperthyroidism (over-active thyroid)
Hypothyroidism (under-active thyroid)
Growth hormone deficiency
Other: *(if 'Other' is selected, please specify in free text)*

Has your relative been diagnosed with any disorder of the endocrine or metabolic systems? *(if 'yes', specify)*

If applicable, what was the age at first menstruation? *(In years)*

45/46. Blood and immune system problems:
Age (by default current age) *(In years)*

Does / did your relative have recurrent infections?
Yes, but only ear / nose / throat infections
Yes *(if 'yes', specify)*
No
I don't know

Has your relative been diagnosed with any disorder of the blood or immune system?

46/46. Cancer development:
Age (by default current age) *(In years)*

Yes / No / I don't know *(Single Choice)*
(if 'yes', specify)
